# The Genomic Consultation Service: A clinical service designed to improve patient selection for genome‐wide sequencing in British Columbia

**DOI:** 10.1002/mgg3.410

**Published:** 2018-05-30

**Authors:** Alison M. Elliott, Christèle du Souich, Shelin Adam, Nick Dragojlovic, Clara van Karnebeek, Tanya N. Nelson, Anna Lehman, The CAUSES Study, Larry D. Lynd, Jan M. Friedman

**Affiliations:** ^1^ Department of Medical Genetics Faculty of Medicine University of British Columbia Vancouver BC Canada; ^2^ BC Children's Hospital Research Institute Vancouver BC Canada; ^3^ Collaboration for Outcomes Research and Evaluation (CORE) Department of Pharmaceutical Sciences University of British Columbia Vancouver BC Canada; ^4^ Department of Pediatrics Centre for Molecular Medicine and Therapeutics Vancouver BC Canada; ^5^ Department of Pediatrics Department of Clinical Genetics Academic Medical Centre Amsterdam The Netherlands; ^6^ Department of Pathology and Laboratory Medicine Faculty of Medicine University of British Columbia Vancouver BC Canada; ^7^ Department of Pathology and Laboratory Medicine BC Children's Hospital Vancouver BC Canada; ^8^ CHEOS – Centre for Health Evaluation and Outcomes Sciences Providence Health Research Institute Vancouver BC Canada

**Keywords:** genetic counselors, genome‐wide sequencing, health services implementation, intellectual disability

## Abstract

**Background:**

Access to clinical diagnostic genome‐wide sequencing (GWS; exome or whole genome sequencing) is limited in British Columbia. The establishment of a translational research initiative (CAUSES) to provide diagnostic genome‐wide sequencing for 500 children necessitated the development of a genomic consultation service, a clinical service established to provide consultation for physicians considering GWS for their pediatric patients throughout British Columbia. The Genomic Consultation Service provides patient‐specific genomic advice that may include: GWS, multi‐gene panel, single gene test, referral to medical genetics for clinical evaluation, or no genetic testing. Here, we describe and evaluate this service.

**Methods:**

We analyzed referral patterns, patient demographics, clinical indications, and genomic advice provided during the first year of this service. Comparison of outcomes from the first 6 months versus the last 6 months was performed.

**Results:**

A total of 407 referrals (238 males and 169 females [*p* = .0006]) were processed in the first year. Only children were eligible for referral and average patient age was 8 years. Medical genetics was the most frequent referring discipline, followed by biochemical disease and pediatric neurology, respectively. Most patients (68%) had syndromic intellectual disability. There was a significant difference in the frequency of referrals not appropriate for GWS in the first versus the second 6 months of the service (75/220 vs. 42/187; *p* = .01) suggesting increasing awareness of testing criteria by referring physicians.

**Conclusion:**

This triage service is utilized throughout the province and appears to be an important factor in the high diagnostic rate (>40%) achieved in our GWS program.

## 1 INTRODUCTION

Clinical diagnostic genome‐wide sequencing (GWS; exome sequencing or whole genome sequencing) is not routinely available in British Columbia, Canada. Although universal medical care is available through the provincial Medical Services Plan (Ministry of Health), funding for genetic testing is limited and is granted only in restricted situations. The CAUSES (Clinical Assessment of the Utility of Sequencing and Evaluation as a Service) Study was established as a 3‐year translational research initiative to perform diagnostic genome‐wide sequencing of 500 British Columbia children with suspected genetic disorders. In order to maximize patient benefits resulting from the study, a clinical Genomic Consultation Service was developed at BC Children's and Women's Hospitals, tertiary care centers providing academic clinical care to the province. The CAUSES Study (a trio‐based research study) considers patients for whom clinical diagnostic GWS is indicated and has a diagnostic rate of >40% (Dragojlovic et al., 2018). The Genomic Consultation service reviews and triages referrals and provide genomic advice for BC physicians considering GWS for their pediatric patients.

The Genomic Consultation Service was established May, 2015, and meets weekly to triage and discuss patient referrals. A one‐page referral form is completed and sent with a copy of the most recent/relevant consultation letter by the referring physician. A clinical team comprised of medical geneticists, a pediatric subspecialist, molecular geneticist, and genetic counselors reviews referrals and related health records and provides advice in a letter to the referring physician. The letter becomes part of the child's health record.

To evaluate the service's performance, we reviewed referral patterns, patient characteristics, and genomic advice during its first year of operation. Comparison of the first 6 months to the second 6 months was performed with respect to genomic advice. Impact on the diagnostic rate in the CAUSES Study and health implementation of GWS was evaluated.

## 2 MATERIALS AND METHODS

### 2.1 Education sessions

**Figure 1:**
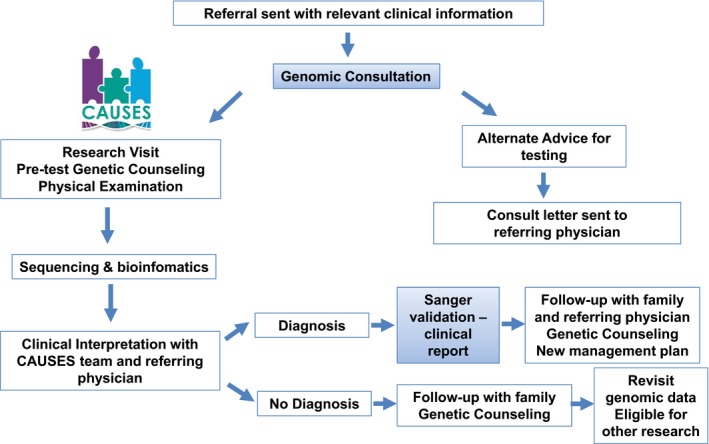
CAUSES research clinic algorithm

Educational sessions were delivered to various pediatric subspecialists, pediatric residents, medical genetics physicians, and genetic counselors prior to the launch of the Genomic Consultation Service and the CAUSES Study. An initial session was conducted for all division heads within the department of pediatrics. At the initial education session, the referral process for the Genomic Consultation (form available online) and the CAUSES research clinic were presented (Figure 1).

Following the initial meeting, each division appointed a representative who would serve as a spokesperson for their section, attend results discussions when the referring physician was not available, and bring suggestions and concerns to the Genomic Consultation and CAUSES Study teams. The Provincial Medical Genetics Program appointed a clinical geneticist who was independent of the CAUSES Study. A follow up education session was conducted for all appointed representatives where the CAUSES Study was explained in detail and the inclusion and exclusion criteria established for GWS were reviewed. Additional education sessions were also conducted including pediatric resident rounds and clinical genetics rounds (with clinical geneticists and genetic counselors in attendance) in order to inform potential referring clinicians.

The Genomic Consultation Service meets weekly to triage and discuss approximately 10 patient referrals. Genetic counselors review each patient chart for detailed phenotypic information and lead the discussion of each referral with the team. The team is comprised of medical geneticists, a pediatric subspecialist, molecular geneticist, and genetic counselors. Advice on the most appropriate testing strategy is included in a letter to the referring physician for the health record. Referring physicians who disagree with the CAUSES team's decision can request reconsideration.

### 2.2 Inclusion and exclusion criteria

As the CAUSES Research Clinic provides trio‐based GWS, availability of both biological parents is required for enrollment. The inclusion criteria are as follows:

- Age ≤19 years
- Patient has a suspected single‐gene disorder plus one or more of the following:
  ○ Previous genetic investigations including chromosomal microarray analysis, appropriate single‐gene or available panel testing, and TIDE first tier biochemical testing for intellectual disability (van Karnebeek, Shevell, Zschocke, Moeschler, & Stockler, 2014) have not identified the genetic cause
  ○ The condition exhibits extensive genetic heterogeneity
  ○ The family history suggests a Mendelian single‐gene disorder (e.g., affected parent and child, unaffected parents and affected child, parental consanguinity, multiple affected siblings, etc.).

Examples of circumstances when a patient would not be enrolled in the CAUSES Study include:

- an infectious, toxic or other nongenetic cause is likely
- a multifactorial condition is likely
- a teratogenic exposure is likely
- a well‐delineated chromosomal disorder was identified in the patient
- a Mendelian condition is suspected with limited genetic heterogeneity for which a targeted (and probably more cost‐effective) single‐gene test or gene panel is available
- the disease is likely to be caused by mutation of a novel human disease gene. (The CAUSES Study is focused on the evaluation of diagnostic GWS as a clinical service, not novel gene discovery).

### 2.3 Health implementation analysis

We tracked the number of referrals, genomic advice provided, and the time required for review of patient records by the genetic counselors prior to the triage meeting and the time required for patient discussion.

### 2.4 Patients

This study was performed at the BC Children's and Women's Hospitals. The Research Ethics Board at UBC/BC Children's and Women's Hospital approved this study (H16‐02645).

### 2.5 Patient demographics

Sex and the age of the patient were recorded. For analysis, age was divided into the following categories: Infant (<1 year); Preschool (>1–4 years); Elementary School (5–11 years); High School/Adolescent (12–19 years).

### 2.6 Indications for diagnostic GWS

For purposes of analysis, the indications for diagnostic GWS were classified into four main categories:

- Isolated (nonsyndromic) Intellectual Disability—patients with intellectual disability and no major congenital anomalies.
- Syndromic Intellectual Disability—patients with intellectual disability and major malformations or multiple minor congenital anomalies or multi‐system medical issues.
- Multiple Congenital Anomalies without intellectual disability.
- Unexplained disorders of organ dysfunction without intellectual disability, patients with serious disorders of the blood, immune system, liver, kidney, endocrine system, neuromuscular function, or other organ system for which a genetic cause is suspected.

### 2.7 Referring clinicians

The clinical specialties and subspecialties of referring physicians were documented.

### 2.8 Genomic advice

The genomic advice provided in a letter for the health record to the referring physician was classified under one of the following categories:

- Referral to the Provincial Medical Genetics Program for complete, in‐person clinical genetics evaluation is indicated;
- Targeted testing for a specific gene or available multi‐gene panel is indicated;
- GWS is not indicated for clinical purposes, but might be appropriate on a research basis through another study at our center or for investigation of a possible novel genetic cause;
- No further genetic testing is indicated at this time;
- Clinical GWS is indicated. If diagnostic GWS is clinically indicated, the referring physician is informed that she/he can request approval for this test through the Ministry of Health Out‐of‐Province Testing procedure or, if eligible according to the inclusion criteria described above, enroll the patient in the CAUSES Study;
- Defer—pending results of current testing;
- Decline for GWS through the CAUSES Study (e.g., one or both parents unavailable, anticipated low diagnostic yield).

### 2.9 Data analysis

Chi‐square test (to compare sex of referred patients) and Fisher's exact test (genomic advice rates between the first and second 6‐month intervals) were used as descriptive statistics (categories 1–4, 6, 7 vs. category 5) in addition to comparison of appropriateness for clinical GWS of Medical Genetics referrals versus all other disciplines for the first year.

## 3 RESULTS

During its first year, 407 genomic consultation referrals were processed (220 referrals in the first 6 months and 187 in the second 6 months). The lead genetic counselor required an average of 45 min to review each referral in detail and prepare it for presentation to the team. At the Genomic Consultation session, each case took approximately 7 min on average to discuss and determine the most appropriate genomic testing advice.

In total, 169 females (42%) and 238 males (58%; *p* = .0006) were referred. The average patient age was 8 ± 5.1 years, with the largest age group 5–11 years (45%). The majority of referred patients had syndromic intellectual disability (68%; Table 1).

**Table 1:** Demographic information of patients referred for Genomic Consultation (first year)

Patient demographics	*N*	%
Sex		
Male	238	58
Female	169	42
Age		
<1 year	21	5
1–4 years	99	24
5–11 years	182	45
12–19 years	105	26
Indication		
Syndromic intellectual disability	278	68
Unexplained disorders of organ function (no intellectual disability)	51	13
Multiple congenital anomalies (no intellectual disability)	50	12
Isolated intellectual disability	28	7

**Figure 2:**
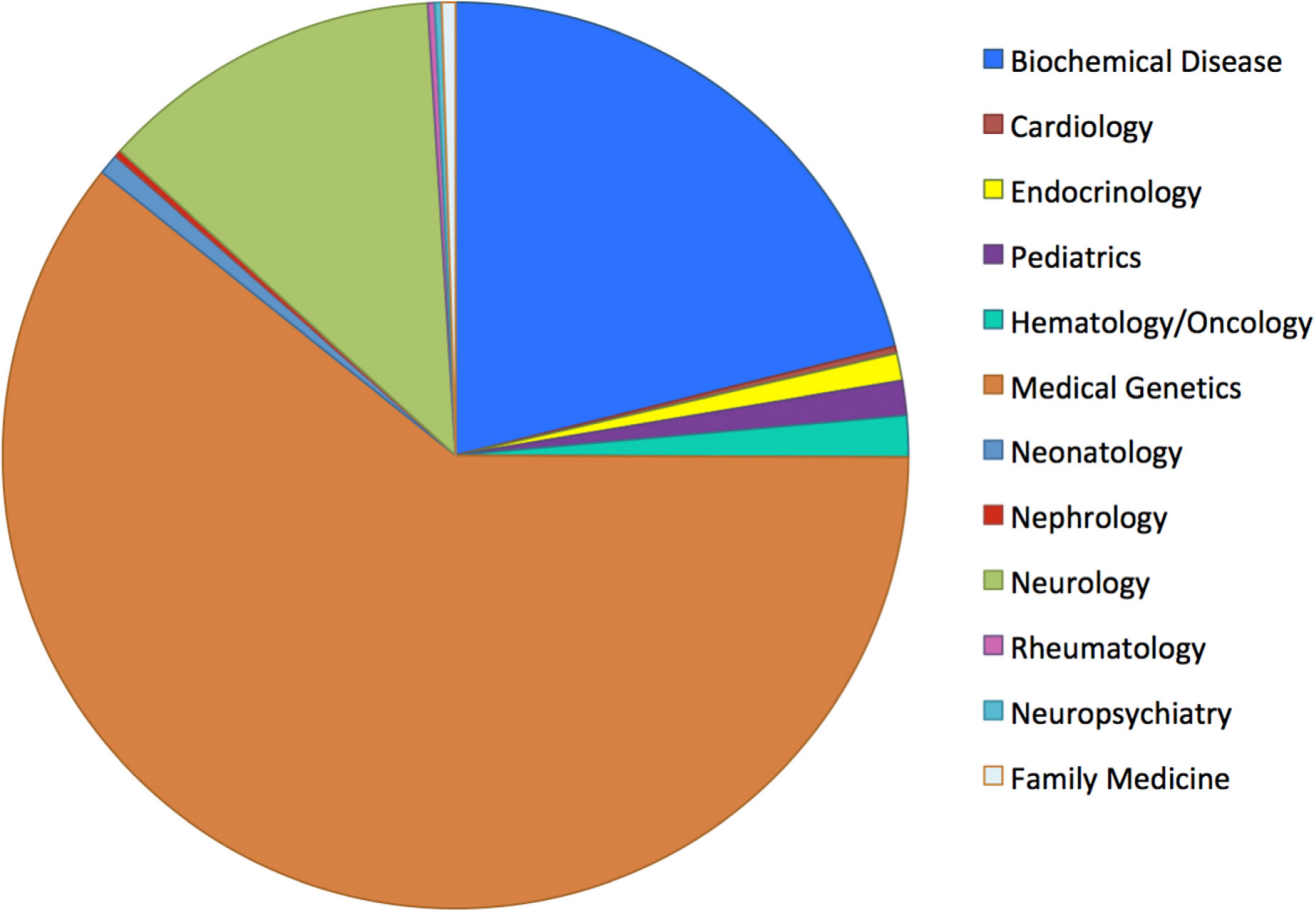
Referring specialties across British Columbia for the Genomic Consultation Service

Medical genetics was the most common referring discipline (61%), followed by metabolic disease/biochemical genetics (21%) and neurology (12%; Figure 2).

**Figure 3:**
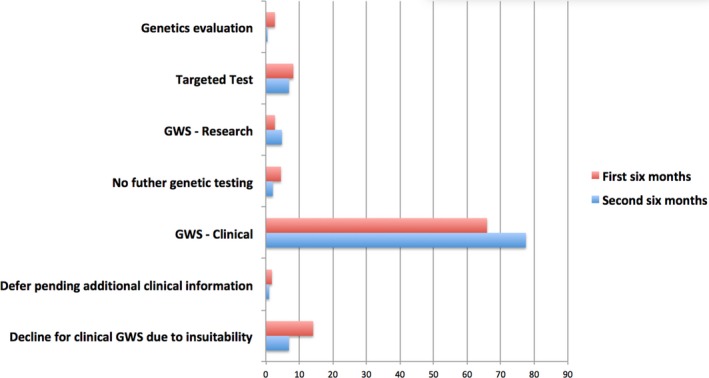
Genomic Advice provided to the referring physicians for patients referred for Genomic Consultation (first 6 months and second 6 months shown, as percentages)

The majority of referrals during the first year of operation of the service (71%) were appropriate for clinical GWS and the CAUSES Study. More referrals not appropriate for clinical GWS through the CAUSES Study were processed during the first 6 months (75/220, 34%) versus the second 6 months of the service (42/187, 22%; *p* = .01; Figure 3). Within British Columbia, many patients undergo GWS as part of research studies. A total of 15 (4%) of genomic consultation referrals were recommended for research GWS projects external to the CAUSES Study. During the first year, 184 (of 247) medical genetics referrals and 106 (of 160) nongenetics referrals were appropriate for clinical GWS and the CAUSES Study (*p* = .07).

## 4 DISCUSSION

Within Canada, access to clinical diagnostic GWS is limited. The Genomic Consultation Service is designed to provide advice regarding appropriate genetic testing for pediatric patients being considered for diagnostic GWS. This service provides clinicians (particularly those external to genetics: pediatric subspecialists, pediatricians, family physicians) an opportunity to interface with a genetics team and receive advice about genetic testing without having to wait many months for a full medical genetics consultation. This service is not a substitute for an in person consultation with a medical geneticist. The majority of patients were referred by medical geneticists in order to obtain diagnostic GWS that was not otherwise available to their patients. Many of the 40% of patients referred by other clinicians had previously been seen in medical genetics as well, which may explain why a medical genetics evaluation was suggested by the Genomic Consultation team in only 2% of referrals. There was not a significant difference between medical genetics and all other disciplines with respect to appropriateness of referral for diagnostic genome‐wide sequencing over the course of the year (*p* = .07).

Guidance on patient selection for diagnostic GWS has been addressed by various authors (ACMG, 2012; Bowdin et al., 2016; Boycott et al., 2015; Matthijs et al., 2016; Ormondroyd et al., 2017; Shashi et al., 2014). Shashi et al. (2014) discussed the necessity of developing clinical guidelines to optimize patient selection for GWS and the importance of the clinical evaluation, which considers the evolving phenotype, emerging diagnostic studies, and benefits and limitations of testing. The Canadian College of Medical Geneticists Position Statement on GWS for monogenic diseases discusses the importance of severity and specificity of patient phenotype, family history, normal chromosomal microarray analysis, exclusion of acquired causes, and clinical interpretation that includes careful patient phenotyping (Boycott et al., 2015). Recent recommendations suggest that a clinician who orders GWS should have knowledge about the test and an opportunity to obtain advice from a genetics professional. At minimum, the necessary knowledge includes the ability to perform a basic clinical genetics evaluation, determine whether GWS is the test of choice for the clinical indication, provide adequate pretest counseling, interpret results, and provide post‐test counseling (Bowdin et al., 2016). Our Genomic Consultation service provides an opportunity for practitioners across British Columbia, including those in remote, rural areas, to access genomic advice and potentially enroll their patients in the CAUSES Study for diagnostic GWS.

The Genomic Consultation Service utilizes genetic counselors to compile extensive patient case histories and streamline case selection. Approximately 45 min were required for case review and preparation per patient by the genetic counselor. A checklist capturing relevant pregnancy, family, developmental and medical histories, key clinical problems, previous consultations and investigations were captured for each patient. The one‐page referral form for physicians was intended to facilitate the referral process for the physician considering GWS for her/his patient. The physician was required to identify primary medical concerns, motivation for GWS (e.g., management) and relevant previous investigations (e.g., chromosomal microarray). This form was usually accompanied by the most recent and relevant consultation. Medical genetics consultations generally contained a comprehensive investigation list, but there was variability in the information provided from physicians. As a result, many referrals required extensive medical chart review. An electronic health record would have facilitated the time required for genetic counselor medical record review and preparation.

Although genetic counselors have the training and expertise to triage genetic testing requests and identify the most appropriate genetic test for a variety of clinical indications, they are not always integrated into the process. Genetic counselors have also been used in the Center for Individualized Medicine at the Mayo Clinic to identify patients suitable for diagnostic GWS (Lazaridis et al., 2014).

This service improves the cost efficiency of GWS as a diagnostic test by utilizing genetic counselors (instead of MD clinical geneticists) to review and prepare referrals for patients being considered for genomic testing, particularly from nongeneticist specialists. Utilization of genetic counselors in the triage processes for genetic testing has been shown to have substantial cost efficiencies. A case study involving one genetic services laboratory demonstrated that integration of genetic counselors into the triage process generated cost savings of more than $1,000,000 through the cancelation of misordered tests over 21 months (Miller et al., 2014). Similarly, integration of a genetic counselor in daily order review and guidance for genetic and genomic testing resulted in the avoidance of $820,887 in excess costs over 26 months in a large academic health care system (Riley, Procop, Kottke‐Marchant, Wylie, & Lacbawan, 2015). There was customized communication with ordering clinicians and tests were approved, canceled, or modified depending on the collaborative decision with the clinician. Moreover, implementation of a combined complex test review and genetic consultation service (with a genetic counselor performing the initial review) within a clinical center has been shown to reduce the number of inappropriate tests, shorten time to diagnosis, and exert a positive influence on health care provider's behaviors (Suarez, Yu, Downs, Costa, & Stevenson, 2017). A multidisciplinary approach to triage patients for GWS similar to the Genomic Consultation Service has also been used elsewhere, although the impact of triage on the cost efficiency of GWS was not evaluated in those cases (Bowdin et al., 2016; Fokstuen et al., 2016; Lazaridis et al., 2016; Ormondroyd et al., 2017). The average presentation time per patient was 7 min. Certain cases required less time when the clinical presentation and key relevant investigations were highly suggestive of the need for GWS (e.g., severe intellectual disability, normal chromosomal microarray, normal MRI, and normal TIDE Tier‐1 investigations).

In line with the utilization management studies discussed above, we have previously shown potential cost efficiencies resulting from the Genomic Consultation Service (Dragojlovic et al., 2018). A costing study conducted as part of the evaluation of the CAUSES Clinic indicated that the Genomic Consultation Service accounted for 15% of the total cost of delivering diagnostic GWS through CAUSES. In a scenario analysis, the estimated per‐patient cost of delivering GWS through CAUSES without the Genomic Consultation Service was lower but the estimated decrease in diagnostic yield that would result from sequencing inappropriate patients without the Genomic Consultation Service increased the estimated cost per positive diagnosis from $14,405 to $15,495 (Dragojlovic et al., 2018).

There is interprovincial variability within Canada with respect to the handling of physician requests for GWS. In some provinces, (e.g., British Columbia), an application is submitted directly to the Ministry of Health (Medical Services Plan) with justification (regarding predicted impact on medical management). In other provinces, there are institutional committees that determine whether GWS will be funded. The concept of an institutional gatekeeper has been discussed (Bowdin et al., 2016; Manolio et al., 2013; Ormondroyd et al., 2017), and the Genomic Consultation Service could provide a potential model for broader implementation. Given that this service was utilized by physicians not only practicing at BC Children's and Women's Hospital, but also those in more remote regions (e.g., Vancouver Island and rural British Columbia, data not shown), demonstrates the potential for uptake throughout the province for a centralized process with the eventual clinical implementation of genome‐wide sequencing.

Our experience indicates that the educational aspects of the Genomic Consultation Service were valuable and should be part of an institutional review committee's operations.

The inclusion/exclusion criteria for clinical GWS (CAUSES Study) determined by our group were consistent with the literature and were described in education sessions and correspondences to referring physicians. Education of referring physicians with ongoing communication is an important consideration with clinical implementation of GWS (Bowdin et al., 2016; Feero & Green, 2011; Lazaridis et al., 2014). The sessions at BC Women's and Children's Hospitals were well attended and all information was distributed electronically following each meeting and subsequently available online via the BC Children's Hospital Research Institute website.

The Genomic Consultation team's decision followed the inclusion/exclusion guidelines with the primary consideration being the likelihood of identifying an underlying Mendelian disorder(s). The need for appropriate initial investigations (e.g., chromosomal microarray analysis for patients with intellectual disability) was transmitted to physicians as part of the educational sessions, and only 1% of total of referrals were deferred due to inadequate clinical information/investigations. Moreover, while approximately 11% of patients were declined for clinical GWS in the CAUSES Study due to lack of suitability of referral, more referrals were deemed inappropriate for clinical GWS during the first 6 months (75) than in the second 6 months of the service (42) (*p* = .01). This difference may be due to learning and increased awareness of patient suitability by referring physicians.

Analysis of patients referred to the Genomic Consultation Service revealed significantly more males than females, which is consistent with other studies involving patients undergoing GWS (Lee et al., 2014; Tarailo‐Graovac et al., 2016; Yang et al., 2014). This is primarily due to the majority of patients having intellectual disability (ID), which is more common in males given the high number of X‐linked genes.

Since confirmation of ID in patients usually occurs after 5 years of age, and ID was the highest indication for referral, the largest age group referred to the Genomic Consultation Service (5–11 years) is not surprising. Idiopathic developmental delay/ID is the most common pediatric referral to medical genetics and is an indication for GWS (Deciphering Developmental Disorders Study, 2017; Elliott et al., 2012; Lee et al., 2014; du Souich et al., 2016; Tarailo‐Graovac et al., 2016; Wright et al., 2015; Yang et al., 2014). The majority of genomic consultation referrals had ID (75%; 68% had syndromic ID, while 7% had nonsyndromic ID). Twenty five percent of patients did not have ID (13% had disorders of organ dysfunction and 12% had multiple congenital anomalies without ID). Each category is an indication for clinical GWS. Currently, these are accepted clinical indications for conventional genetic testing (e.g., chromosomal microarray analysis or targeted genetic testing), but these other forms of genetic testing are less informative than GWS. The majority (71%) of referred patients were appropriate for clinical GWS over the course of the year. Targeted genetic testing (e.g., multi‐gene panel) was recommended in 8% of referrals. Specific multi‐gene panel recommendations were included in the letter to the referring physician and could be used to support applications to the Medical Service Plan for funding of the test. However, if the panel was more costly than clinical GWS, the patient was classified as appropriate for clinical GWS. This is similar to the triage strategy adopted at University Hospitals of Geneva, where costing is a component of the consideration (Fokstuen et al., 2016). Patients eligible for high‐throughput sequencing included those with a likely heterogeneous Mendelian disorder with at least one known clearly pathogenic gene and patients with developmental delay of unknown origin, but the cost of performing high‐throughput sequencing needed to be less than the Sanger sequencing of the corresponding genes (Fokstuen et al., 2016).

The Genomic Consultation Service likely contributed to improving the diagnostic yield and appropriate use of GWS in our patients suspected of having rare genetic diseases. The Genomic Consultation Service was utilized by physicians throughout British Columbia who were considering GWS for their patients. We did not demonstrate a significant difference between referrals from medical genetics versus other disciplines for appropriateness for clinical GWS and the CAUSES Study. We have also demonstrated that appropriate patient selection through this service improves the cost efficiency of delivering a diagnostic GWS program (Dragojlovic et al., 2018). The Genomic Consultation Service is a successful approach that enriches the selection of patients suitable for GWS (i.e., with monogenic disease) and provides physicians (particularly those external to medical genetics) with patient‐specific genomic advice, which can support diagnostic care and applications to the Medical Service Plan (Ministry of Health). Furthermore, this service provides an accessible and effective model for the eventual provincial rollout of clinical GWS.

## CONFLICT OF INTEREST

None declared.
